# Three-dimensional assessment of periodontal support of lower incisors for skeletal Class II malocclusion undergoing presurgical orthodontic treatment with different vertical skeletal patterns

**DOI:** 10.1186/s40510-023-00495-y

**Published:** 2023-12-18

**Authors:** Hangmiao Lyu, Huimin Ma, Xiaoxia Wang, Li Xu, Jianxia Hou, Yijiao Zhao, Weiran Li, Xiaotong Li

**Affiliations:** 1 Department of Orthodontics, National Engineering Laboratory for Digital and Material Technology of Stomatology, Beijing Key Laboratory of Digital Stomatology, Peking University School and Hospital of Stomatology, 22 Zhongguancun Avenue South, Haidian District, Beijing, 100081 People’s Republic of China; 2 Department of Oral and Maxillofacial Surgery, National Engineering Laboratory for Digital and Material Technology of Stomatology, Beijing Key Laboratory of Digital Stomatology, Peking University School and Hospital of Stomatology, 22 Zhongguancun Avenue South, Haidian District, Beijing, 100081 People’s Republic of China; 3 Department of Periodontology, National Engineering Laboratory for Digital and Material Technology of Stomatology, Beijing Key Laboratory of Digital Stomatology, Peking University School and Hospital of Stomatology, Haidian District, Beijing, 100081 People’s Republic of China; 4 Center of Digital Dentistry, Peking University School and Hospital of StomatologyNational Engineering Laboratory for Digital and Material Technology of StomatologyResearch Center of Engineering and Technology for Digital Dentistry of Ministry of HealthBeijing Key Laboratory of Digital Stomatology, Peking University School and Hospital of Stomatology, 22 Zhongguancun Avenue South, Haidian District, Beijing, 100081 People’s Republic of China

**Keywords:** Cone-beam computed tomography, Three-dimensional reconstruction, Alveolar bone remodeling, Mandibular incisor retraction

## Abstract

**Background:**

The aim of the present study was to compare periodontal support changes during retraction of mandibular anterior teeth for skeletal Class II malocclusion with different facial divergence and to analyze relevant factors influencing bone remodeling by applying three-dimensional (3D) cone-beam computed tomography (CBCT) reconstruction technology.

**Methods:**

Forty-eight patients with Class II malocclusion requiring surgical orthodontic treatment enrolled in the study were divided into the hyperdivergent group (*n* = 16), normodivergent group (*n* = 16) and hypodivergent group (*n* = 16) according to their vertical skeletal patterns. Cone-beam computed tomography (CBCT) scans were obtained before treatment (T1) and after presurgical orthodontic treatment (T2). The two-dimensional (2D) alveolar bone morphology, movement of mandibular central incisors and volume of the alveolar bone around incisors were measured on the labial and lingual sides by 3D CBCT reconstruction technology. Statistical analyses were performed with one-way ANOVA, paired t tests and multiple linear regression.

**Results:**

During presurgical orthodontic treatment, the alveolar bone height on the labial side of the hyperdivergent group decreased significantly (*P* ≤ 0.05), but was maintained in the normodivergent and hypodivergent groups (*P* > 0.05). However, the alveolar bone volume, alveolar bone thickness at each level and alveolar bone height on the lingual side decreased significantly for all the groups. Apart from the initial morphometric measurements at T1, the morphology of lingual alveolar bone at T2 was significantly influenced by the direction and amount of tooth movement. Horizontal retraction and vertical protrusion of the root apex were negatively related to the alveolar bone on the lingual side after presurgical orthodontic treatment.

**Conclusion:**

For Class II malocclusion patients undergoing presurgical orthodontic treatment, the changes in the periodontal support of the lower central incisors varied in different vertical skeletal patterns. There exists a great periodontal risk of alveolar bone resorption on the lingual side for various vertical types. To avoid alveolar bone deterioration, it is essential to investigate the bone remodeling of patients with different alveolar bone conditions and cautiously plan tooth movement prior to orthodontic treatment. Moreover, 3D measurements based on CBCT construction can provide complementary information to traditional 2D measurements.

## Background

Adults with severe skeletal Class II malocclusion need surgical orthodontic treatment to improve occlusal function and esthetic appearance [1]. During decompensation treatment before orthognathic surgery, mandibular incisors must undergo retraction [2]. Anatomically, the alveolar bone becomes thinner from the posterior to the anterior region in the mandible. Mandibular incisors are the ones most prone to gingival recession, dehiscence and fenestrations [3, 4]. For patients with bialveolar protrusion, excessive retraction of mandibular anterior teeth with premolar extraction increases the risk of periodontal deterioration [5, 6], and 48% of lingual sides of mandibular central incisors exhibit a bone height decrease of > 2 mm [7]. On the other hand, it was demonstrated that, under some circumstances, an increase in labial alveolar bone due to orthodontic mandibular incisor retraction can be expected [8]. A previous study indicated that Class II patients had a greater prevalence of fenestration than Class I and Class III malocclusions [9]. Moreover, skeletal Class II patients have thinner labial cortical bone in mandibular anterior teeth than skeletal Class I patients [10], which demonstrates periodontal risks in the presurgical decompensation phase of mandibular incisors. Hence, it is crucial to evaluate mandibular alveolar bone loss in orthodontic patients with skeletal Class II occlusion during presurgical orthodontic movement.

Alveolar bone condition after orthodontic treatment is dominantly influenced by the morphology of alveolar bone before orthodontic treatment and bone remodeling secondary to orthodontic tooth movement [11]. Previous studies have reported that vertical skeletal patterns are important factors influencing the morphology of alveolar bone. Hyperdivergent subjects have thinner cortical alveolar bone than normodivergent and hypodivergent subjects [10, 12, 13]. The lingual alveolar bone thickness of mandibular anterior teeth was negatively correlated with the FMA value [14]. Furthermore, it is widely accepted that orthodontic movement should allow the tooth to remain within the bone. Proffit proposed the 'envelope of discrepancy' to represent the limits of tooth movement [15]. Violating the bone envelope can lead to adverse reactions such as bone dehiscence, gingival recession and root resorption [16–19]. Therefore, the vertical skeletal patterns and direction and amount of orthodontic movement should be thoroughly considered when performing en masse retraction of mandibular incisors.

Due to the ability to evaluate the height and thickness of the alveolar bone and the orthodontic movement of every single tooth, cone-beam computed tomography (CBCT) has become increasingly mainstream in diagnostics and orthodontic treatment planning [20]. In addition to two-dimensional (2D) linear measurement in the sagittal plane, CBCT has made it possible to assess alveolar bone condition in a more intuitive and comprehensive way. The periodontal ligament area and the alveolar bone volume of different surfaces around the tooth could be measured by three-dimensional (3D) CBCT reconstruction technology [21–23]. Previous studies have reported that digital teeth and bone models generated by 3D CBCT reconstruction can provide accurate and reliable information compared with microcomputed tomography (micro-CT) data [24–26]. Earlier research also found that CBCT with a voxel size of 0.30 mm reliably provided accurate data regarding the alveolar crest morphology [22]. Moreover, previous studies have found that the 3D measurement of root surface area of periodontal attachment and 2D vertical bone level is inconsistent [27], and diagnosis on the basis of 2D radiographic bone without considering the 3D condition may underestimate the severity of periodontal damage [28].

At present, previous studies have mainly focused on the morphologic changes in alveolar bone during lower incisor retraction without considering the influence of vertical skeletal patterns on alveolar bone conditions. Furthermore, the association between tooth movement and remodeling of the surrounding bone is still controversial. To avoid alveolar bone deterioration, it is essential to investigate the bone remodeling of patients with different alveolar bone conditions and cautiously plan tooth movement prior to orthodontic treatment. Hence, this study aimed to compare alveolar bone changes during retraction movement of mandibular anterior teeth with premolar exaction for skeletal Class II malocclusion with different facial divergence and to analyze relevant factors influencing bone remodeling.

## Material and methods

### Selection of the sample

This retrospective cohort study was approved by the Biomedical Ethics Committee. Forty-eight patients with Class II malocclusion requiring surgical orthodontic treatment were enrolled in the study from 2014 to 2022. These subjects were divided into 3 groups according to their vertical skeletal patterns (Table 1): Group 1 comprised 16 patients (8 men, 8 women; age, 25.81 ± 4.90 years) with a hyperdivergent skeletal pattern [SN-MP (°) > 37.7°]; group 2, 16 patients (8 men, 8 women; age, 26.31 ± 5.74 years) with a normodivergent skeletal pattern [27.3° < SN-MP (°) ≤ 37.7°]; and group 3, 16 patients (8 men, 8 women; age, 23.75 ± 4.70 years) with a hypodivergent skeletal pattern [SN-MP (°) < 27.3°].

**Table 1:** Baseline patient characteristics

Variables	Group 1	Group 2	Group 3	*P*	Multiple comparisons
Age, y	25.81 ± 4.90	26.31 ± 5.74	23.75 ± 4.70	0.335	–
ANB (°)	8.37 ± 1.46	8.28 ± 1.64	7.18 ± 1.40	0.192	–
SN-MP (°)	42.64 ± 3.46	33.99 ± 1.62	25.73 ± 3.32	0.000**	1 > 2 > 3
L1-MP (°)	96.88 ± 6.80	101.08 ± 4.48	108.63 ± 6.49	0.000**	3 > (1,2)
Duration, mon	28.19 ± 5.92	27.31 ± 7.61	29.81 ± 7.24	0.592	–

P, One-way ANOVA with Duncan's multiple comparison test was performed for comparisons among group 1, group 2 and group 3. Results are expressed as mean ± standard deviation

*P ≤ 0.05; **P ≤ 0.01

Inclusion criteria were as follows: (1) age > 18 years; (2) skeletal and dental Class II malocclusion (ANB angle > 4.7°; overjet > 4 mm); (3) mild crowding (< 4 mm) in the mandibular arch and the initial rotation degrees of mandibular central incisors < 15°[29]; and (4) presurgical orthodontic design as follows: Mandibular bilateral first premolars were extracted, and anchorage in the mandibular arch was reinforced using miniscrew implants.

The exclusion criteria were severe facial asymmetry (> 3 mm of chin point deviation from the facial midline), poor oral hygiene and uncontrolled periodontal disease, cleft lip or palate or other craniofacial syndromes, missing or decayed teeth before treatment (except for the third molars) and orthodontic treatment history.

All orthodontic treatments were performed by a single orthodontist with a straight-wire fixed appliance (0.022-in slot size, MBT prescription), and the archwire sequence involved 0.014-, 0.016-, 0.018- and 0.018 × 0.025-in nickel–titanium wires followed by a 0.018 × 0.025-in stainless steel wire. After presurgical orthodontic treatment, all the subjects underwent bilateral sagittal split ramus osteotomy and LeFort I surgery with rigid internal fixation.

CBCT scans were obtained before treatment (T1) and after presurgical orthodontic treatment (T2). CBCT images were acquired with the New Tom VG device (Aperio Services, Italy) at the following settings: 3.0 mA, 110 kV, exposure time of 1.8 s, voxel size of 0.25 mm and scanning area of 10 × 10 cm.

Lower central incisors (LCIs) on the right side were selected as subjects for measurement. The definitions of the measurements used in this study are described in Table 2. The measurements in this study were modified based on those reported by Lee et al. [30] and Ma et al. [31]

**Table 2:** Definition of measurements

Measurement type	Definition
V-LA, mm^3^	Labial surrounding alveolar bone volume
V-L, mm^3^	Lingual surrounding alveolar bone volume
V-W, mm^3^	Whole surrounding alveolar bone volume
PDLA, mm^2^	The part of the root surface area that is covered by PDL and alveolar bone
RL, mm	Root length
T-ALA, mm	Labial alveolar bone thickness at apex level
T-AL, mm	Lingual alveolar bone thickness at apex level
T-AW, mm	Whole alveolar bone thickness at apex level
T-MLA, mm	Labial alveolar bone thickness at middle root level
T-ML, mm	Lingual alveolar bone thickness at middle root level
T-MW, mm	Whole alveolar bone thickness at middle root level
VBL-LA, mm	Distance from CEJ to alveolar crest on the labial side parallel to root length
VBL-L, mm	Distance from CEJ to alveolar crest on the lingual side parallel to root length
VBL-M, mm	Distance from CEJ to alveolar crest on the mesial side parallel to root length
VBL-D, mm	Distance from CEJ to alveolar crest on the distal side parallel to root length

### Alveolar bone volume measurements

Digital Imaging and Communication in Medicine (DICOM) files of CBCT were imported into Mimics 19.0 software (Materialise, Leuven, Belgium). In Mimics, 3D digital models of the lower central incisors and bone in vivo were reconstructed on the basis of those reported by Forst et al. [32] and Lyu et al. [21, 22]

The digital models were exported in stereolithographic (STL) format and imported into Geomagic software (Geomagic, Cary, NC, USA). The measurements of alveolar bone volume in this study were modified on the basis of those reported by Zhang et al. [23].

**Fig. 1:**
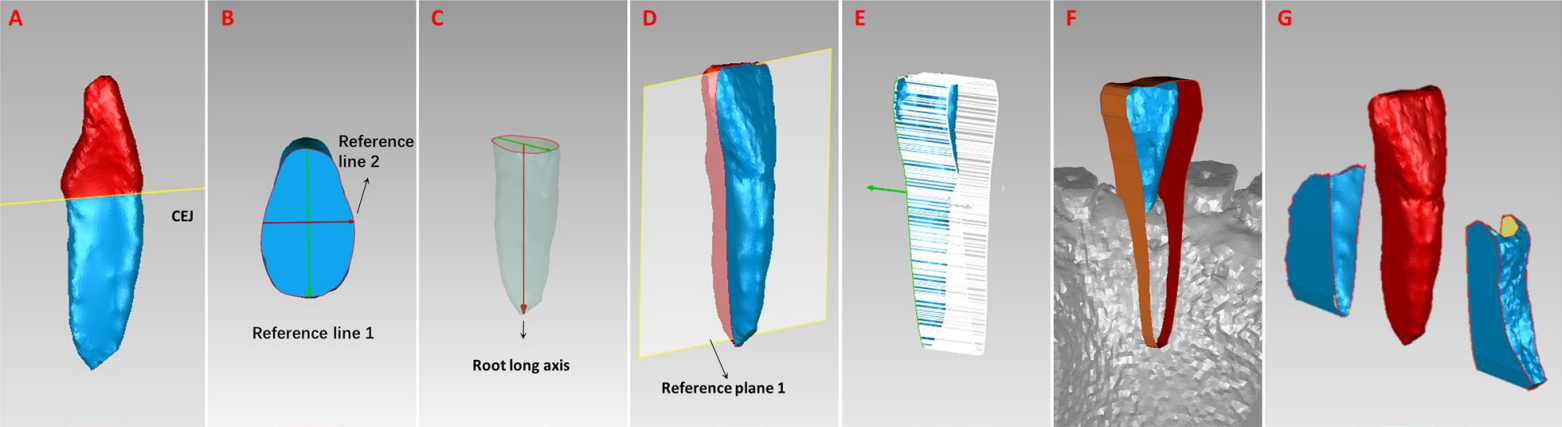
Alveolar bone volume measurements. **A,** The tooth model was cut along the cementoenamel junction (CEJ) plane. **B,** On the CEJ plane, the widest labiolingual distance was defined as reference line 1. Reference line 2 was a line perpendicular to reference line 1 through the midpoint of reference line 1. **C,** The line from the midpoint of line 1 to the root apex point was the root long axis. **D,** Reference plane 1 was formed by reference line 2 and the root long axis. The tooth model was separated into the labial side and lingual side by reference plane 1. **E,** The tooth contour boundary lying on plane 1 was projected perpendicular to plane 1 toward the lingual side using the extrude boundary method. **F,** The lingual alveolar bone volume model was cut out of the bone digital model by the extruding boundary. **G,** An example of labial and lingual alveolar bone volume models

To obtain the labial and lingual alveolar bone volume models, we first cut the tooth model along the CEJ plane and removed the crown in Geomagic (Fig. 1A). On the CEJ plane, the widest labiolingual distance was defined as reference line 1. Reference line 2 was a line perpendicular to reference line 1 through the midpoint of reference line 1 (Fig. 1B). The line from the midpoint of line 1 to the root apex point was the root long axis (Fig. 1C). Reference plane 1 was formed by reference line 2 and the root long axis. The tooth model was separated into two parts by reference plane 1 (Fig. 1D). Then, we projected the tooth contour boundary lying on plane 1 perpendicular to plane 1 using the extrude boundary method (Fig. 1E). Afterward, we cut out the labial and lingual alveolar bone volume models from the bone model using the extruding boundary, respectively (Fig. 1F, G). The measurement of periodontal ligament area (PDLA) was described in detail in previous study [22].

### 2D measurements

**Fig. 2:**
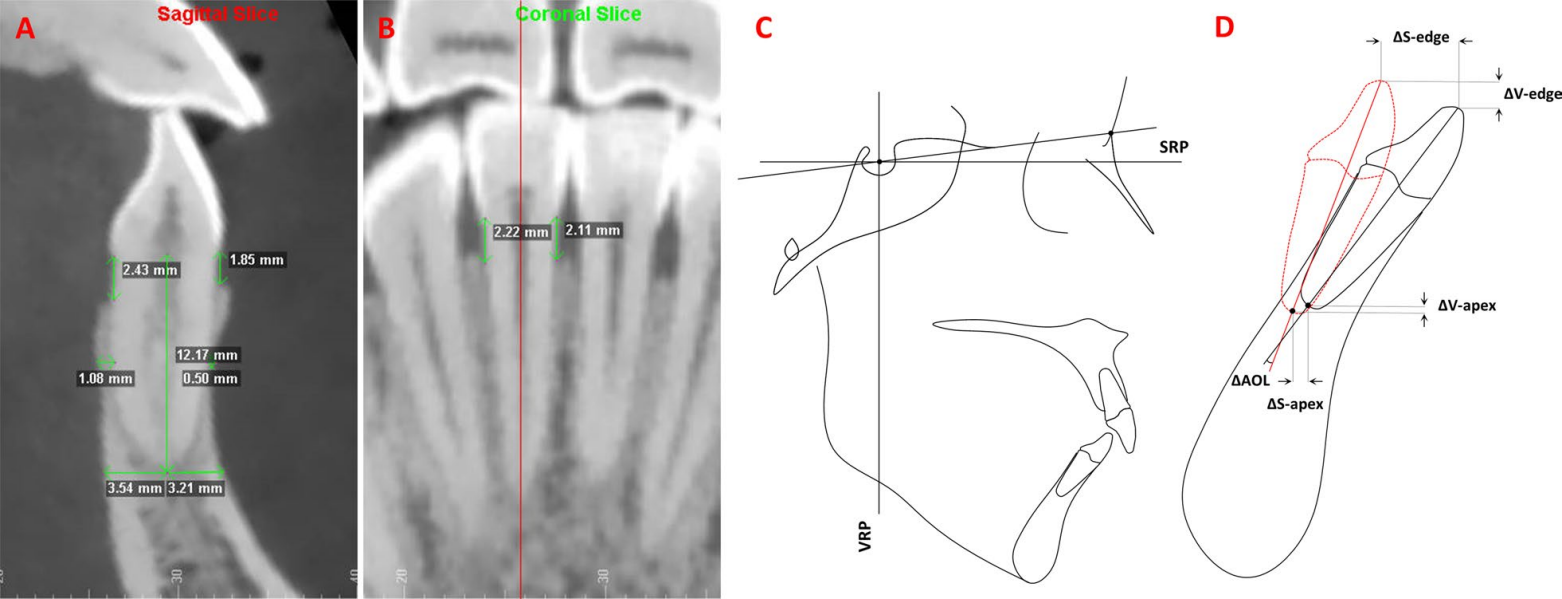
2D measurements of alveolar bone around LCIs and tooth movement. **A,** Measurements of RL, ABT at the apex level and middle root level, and VBLs on the labial and lingual sides in the sagittal slice. **B,** Measurements of VBLs on the mesial and distal sides in the coronal slice. **C,** Reference lines and measurements of LCI movement. Reference lines: SRP, sagittal reference plane, a horizontal plane angulated 7° clockwise to the sella–nasion plane passing through the sella; VRP, vertical reference plane, plane perpendicular to the SRP passing through the sella. **D,** ΔS-edge, movement distance of the incisal edge along the sagittal reference plane (SRP); ΔS-apex, movement distance of the root apex along the sagittal reference plane (SRP); ΔV-edge, movement distance of the incisal edge along the vertical reference plane (VRP); ΔV-apex, movement distance of the root apex along the vertical reference plane (VRP); ΔAOL, the angles of the long axes between T1 and T2

The alveolar bone morphology and root length (RL) of the right LCIs were measured on CBCT images evaluated with Dolphin 11.8 software (Dolphin Imaging & Management Solutions, USA) (Fig. 2). The images were oriented along the root long axis, the line from the midpoint of the cementoenamel junction to the apical point. As shown in Table 2, the specific measurement variables included root length (RL), vertical alveolar bone level (VBL) and alveolar bone thickness (ABT). The ABT was measured at the middle root level and root apex level on the sagittal slices where incisors were the widest labiolingually in the axial view. The VBL included the labial side (VBL-LA), lingual side (VBL-L), mesial side (VBL-M) and distal side (VBL-D).

### Measurements of tooth movement

The distance between the presurgical and surgical orthodontic treatment of LCIs was measured by the superimposition of CBCT images with a voxel-based method [33]. The midsagittal plane was selected after reorientation, and the SN plane was rotated 7° clockwise as the horizontal coordinate axis [34]. The sagittal and vertical distances were then measured between the incisal edge points at T1 and T2 and the root apex at T1 and T2, and the angles of the long axes between T1 and T2 were measured (ΔAOL) (Fig. 2).

The sample size was calculated on the basis of the VBL and ABT reported by Hung BQ et al. [6] With a and power values set at 0.05 and 80%, respectively, 16 samples per group were needed.

### Statistical analysis

Statistical analyses were performed using SPSS (version 20.0; IBM Corp, Armonk, NY). One-way analysis of variance with Duncan’s multiple comparison test was performed to compare measurements among groups at T1. The Mann‒Whitney U test was used to compare the changing amounts of measurements from T1 to T2 among groups. A paired t test was used to compare changes in all the measurements before and after presurgical orthodontic treatment in the 3 groups. One-sample t test was used to compare tooth movement measurements of LCIs and 0 for different groups. At the same time, Pearson correlation and multiple linear regression analyses were performed to analyze alveolar bone morphologic measurements after presurgical orthodontic treatment (T2).

All measurements were taken twice by the same investigator at an interval of 2 weeks. The average value of the 2 measurements was used for statistical analysis. Bland‒Altman tests were applied to compare the 2 measurements. The mean difference between the measures was − 0.01 and − 0.31 for 2D and 3D measurements, respectively. The 95% limits of agreement were [− 0.52, 0.50] for 2D measurements and [− 8.33, 7.72] for 3D measurements. In addition, strong intraexaminer reliability was found for 2D (ICC = 0.996, 95% CI 0.995–0.997) and 3D measurements (ICC = 0.992, 95% CI 0.990–0.993).

## Results

Before treatment, the V-LA of the hypodivergent group was larger than that of the normodivergent group. At the apical apex level, the hypodivergent group had significantly greater T-ALA, T-AL and T-AW than the other 2 groups. At the middle root level, the T-MW and T-ML of the hypodivergent group were significantly greater than those of the hyperdivergent group. However, the RL, PDLA and VBL at the labial, lingual, mesial and distal sides among all three groups were similar, and the T-MLA was approximately thin before treatment (Table 3).

**Table 3:** Comparison of the RL and alveolar bone morphologic measurements in groups from T1 to T2

	Group 1			Group 2			Group 3			Comparison among groups at T1	
Measurements	T1	T2	P1	T1	T2	P2	T1	T2	P3	P4	Multiple comparisons
V-LA, mm^3^	26.22 ± 11.89	22.13 ± 11.96	0.202	22.13 ± 6.61	32.83 ± 20.15	0.040*	30.66 ± 7.85	42.07 ± 25.51	0.076	0.037*	(3,1) > (1,2)
V-L, mm^3^	37.05 ± 16.68	19.72 ± 16.34	0.000**	45.09 ± 17.44	18.39 ± 14.32	0.000**	51.03 ± 16.49	18.81 ± 10.70	0.000**	0.074	–
V-W, mm^3^	63.27 ± 24.51	41.85 ± 20.88	0.000**	67.23 ± 21.14	51.22 ± 20.80	0.010**	81.70 ± 20.64	60.87 ± 28.72	0.002**	0.057	–
PDLA, mm^2^	115.73 ± 25.54	99.46 ± 25.37	0.000**	114.03 ± 14.90	92.95 ± 17.28	0.000**	120.25 ± 19.00	95.72 ± 26.79	0.000**	0.671	–
RL, mm	11.30 ± 1.49	10.07 ± 1.42	0.000**	11.18 ± 1.04	9.90 ± 0.90	0.000**	11.55 ± 1.11	10.02 ± 1.71	0.000**	0.686	–
T-ALA, mm	2.64 ± 0.74	3.49 ± 1.48	0.016*	2.57 ± 0.80	4.14 ± 2.17	0.004**	3.66 ± 1.36	6.06 ± 3.06	0.003**	0.005**	3 > (1,2)
T-AL, mm	3.06 ± 1.08	1.65 ± 1.74	0.001**	4.23 ± 1.16	2.01 ± 1.69	0.000**	4.45 ± 0.93	2.21 ± 1.07	0.000**	0.001**	3 > 2 > 1
T-AW, mm	5.70 ± 0.91	5.14 ± 1.77	0.082	6.79 ± 1.03	6.14 ± 1.71	0.057	8.11 ± 1.44	8.27 ± 2.85	0.755	0.000**	3 > 2 > 1
T-MLA, mm	0.63 ± 0.28	0.79 ± 0.56	0.28	0.68 ± 0.33	1.00 ± 0.83	0.078	0.79 ± 0.26	1.90 ± 1.08	0.001**	0.293	–
T-ML, mm	1.06 ± 0.44	0.25 ± 0.48	0.000**	1.43 ± 0.66	0.47 ± 1.03	0.003**	1.56 ± 0.40	0.15 ± 0.47	0.000**	0.023*	3 > 2 > 1
T-MW, mm	1.69 ± 0.65	1.04 ± 0.50	0.003**	2.12 ± 0.69	1.47 ± 1.22	0.042*	2.35 ± 0.50	2.05 ± 1.11	0.189	0.014*	(3,2) > (2,1)
VBL-LA, mm	2.16 ± 1.24	3.69 ± 2.64	0.021*	2.56 ± 1.63	3.46 ± 2.51	0.211	1.86 ± 0.63	2.42 ± 1.39	0.087	0.285	–
VBL-L, mm	2.37 ± 0.79	7.69 ± 3.05	0.000**	2.28 ± 0.88	6.92 ± 2.99	0.000**	2.23 ± 0.77	7.48 ± 2.44	0.000**	0.886	–
VBL-M, mm	1.93 ± 0.42	2.34 ± 0.40	0.001**	2.35 ± 0.88	2.37 ± 0.68	0.936	1.94 ± 0.62	2.39 ± 0.86	0.029*	0.13	–
VBL-D, mm	2.07 ± 0.54	2.35 ± 0.80	0.056	2.49 ± 0.85	2.42 ± 0.92	0.782	2.09 ± 0.63	2.53 ± 0.83	0.042*	0.161	–

P1, paired-samples t test for comparison between T1 and T2 in the group 1; P2, paired-samples t test for comparison between T1 and T2 in the group 2; P3, paired-samples t test for comparison between T1 and T2 in the group 3; P4, one-way ANOVA with Duncan's multiple comparison test was performed for comparisons among group 1, group 2 and group 3 at T1. Results are expressed as mean ± standard deviation

*P ≤ 0.05; **P ≤ 0.01

**Fig. 3:**
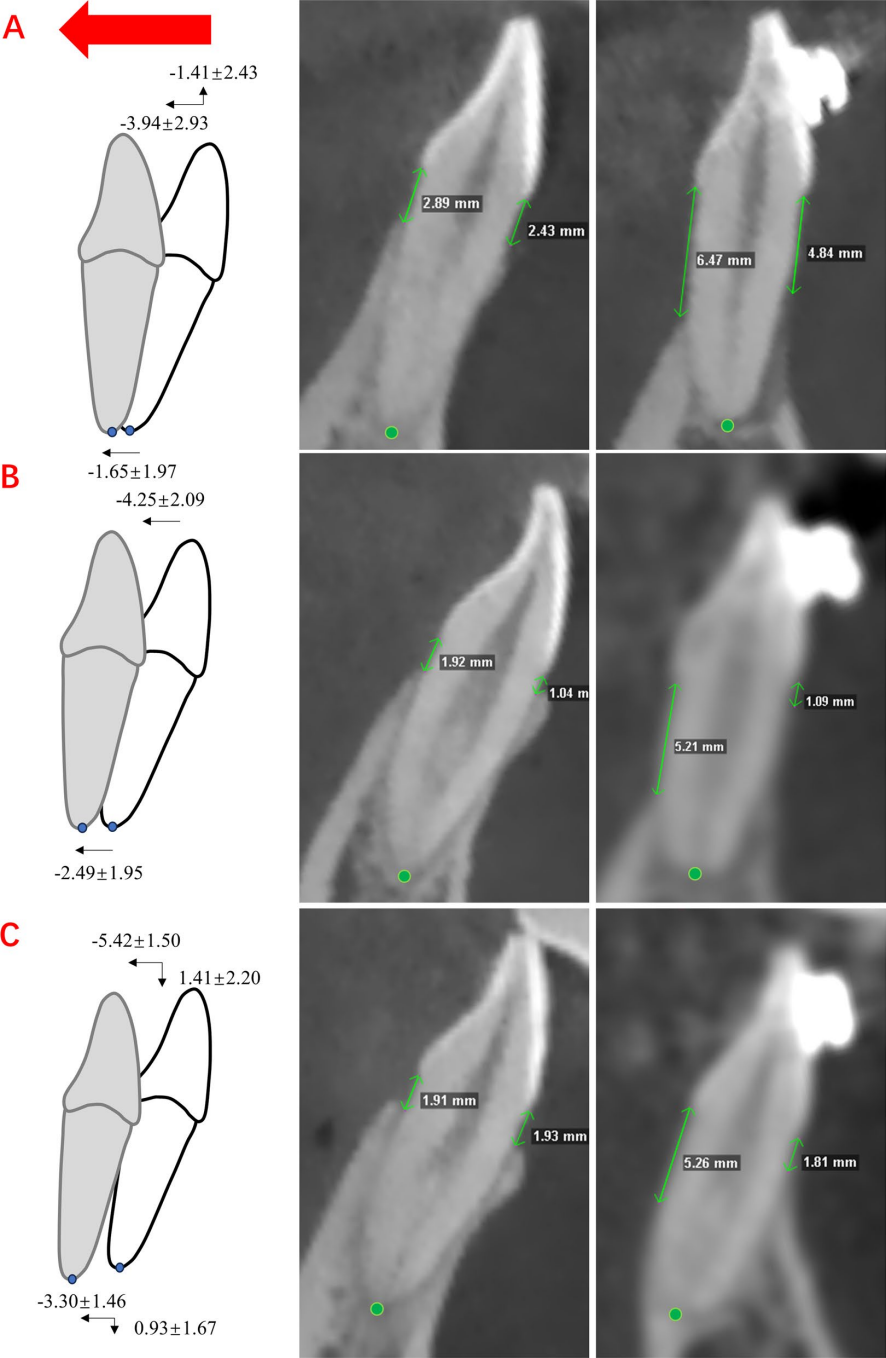
Illustrations and examples of LCI movement (mm) and alveolar bone changes before and after decompensation. **A**, Hyperdivergent group. **B**, Normodivergent group. **C**, Hypodivergent group. Data are the mean ± standard deviation

During the decompensation procedure, the incisal edges among all the groups moved backward horizontally, and there was no significant difference among groups (3.94 mm in the hyperdivergent group, 4.25 mm in the normodivergent group and 5.42 mm in the hypodivergent group on average). The apical points moved backward among all the groups, and the horizontal movement of the hypodivergent group was significantly greater than that of the hyperdivergent group (1.65 mm in the hyperdivergent group and 3.30 mm in the hypodivergent group on average). Vertical changes in the apical point and incisal edge were not obvious in the normodivergent group during presurgical treatment. The incisal edge moved 1.41 mm downward in the hypodivergent group and 1.41 mm upward in the hyperdivergent group on average with statistical significance (Fig. 3). In addition, LCIs in the hyperdivergent group and normodivergent group were lingually inclined by 6.65° and 4.68° on average, respectively, with a significant difference (Table 4).

**Table 4:** Movement of LCIs during decompensation of skeletal Class II malocclusion with different vertical skeletal patterns

Measurements	Group 1	P1	Group 2	P2	Group 3	P3	P4	Multiple comparisons
Δ S-apex	− 1.65 ± 1.97	0.004**	− 2.49 ± 1.95	0.000**	− 3.30 ± 1.46	0.000**	0.045*	(1,2) > (2,3)
Δ V-apex	− 0.42 ± 1.71	0.337	− 0.36 ± 1.27	0.271	0.93 ± 1.67	0.041*	0.029*	3 > (1,2)
Δ S-edge	− 3.94 ± 2.93	0.000**	− 4.25 ± 2.09	0.000**	− 5.42 ± 1.50	0.000**	0.159	–
Δ V-edge	− 1.41 ± 2.43	0.035*	− 0.22 ± 2.23	0.698	1.41 ± 2.20	0.022*	0.005**	(3,2) > (2,1)
Δ AOL	− 6.65 ± 6.22	0.001**	− 4.68 ± 8.07	0.035*	− 3.93 ± 7.91	0.065	0.571	–

Δ S-apex and Δ S-edge, positive values representing forward movement and negative values representing retraction of the root apex and the incisal edge; Δ V-edge and Δ V-apex, positive values representing intrusion and negative values representing the extrusion of the incisal edge and root apex; ΔAOL, positive values indicates proclination and negative values indicates retroclination; P1, one-sample t-test for comparison between measurements and 0 for the group 1; P2, one-sample t-test for comparison between measurements and 0 for the group 2; P3, one-sample t-test for comparison between measurements and 0 for the group 3; P4, one-way ANOVA with Duncan's multiple comparison test was performed for comparisons among group 1, group 2 and group 3. Results are expressed as mean ± standard deviation

*P ≤ 0.05; **P ≤ 0.01

From T1 to T2, the RL in all three groups decreased by approximately 1 mm. On the labial side, the V-LA of all three groups increased or was maintained. The ΔV-LA of the hyperdivergent group was significantly smaller than that of the normodivergent and hypodivergent groups (Table 5). At the root apex level, the alveolar bone thickness of all the groups increased significantly. However, at the middle root level, although the T-MLA of the hypodivergent group increased significantly, there was no significant change in the hyperdivergent and normodivergent groups during the decompensation procedure (Table 3). The ΔT-MLA of the hypodivergent group was also greater than that of the hyperdivergent group (Table 5).

**Table 5:** Comparison of the changes of RL and alveolar bone morphologic measurements (T2-T1) among groups

	Group 1 M(P25, P75)	Group 2 M(P25, P75)	Group 3 M(P25, P75)	*P*	Multiple comparison
V-LA, mm^3^	− 5.97(− 10.08, 4.12)	6.09(− 5.41, 22.77)	10.89(− 8.19, 26.44)	0.04*	(2,3) > 1
V-L, mm^3^	− 16.53(− 24.76, − 7.17)	− 27.80(− 37.97, − 15.00)	− 31.41(− 39.40, − 22.16)	0.02*	(1,2) > (2,3)
V-W, mm^3^	− 15.08(− 28.71, − 9.32)	− 11.73(− 29.47, − 3.70)	− 21.63(− 36.76, − 2.97)	0.68	–
PDLA, mm^2^	− 14.26(− 24.85, − 5.70)	− 21.30(− 29.99, − 8.84)	− 23.40(− 35.63, − 13.53)	0.27	–
RL, mm	− 1.27(− 1.54, − 0.80)	− 1.09(− 2.02, − 0.67)	− 1.46(− 1.71, − 0.72)	0.82	–
T-ALA, mm	0.83(0.30, 1.76)	1.46(0.06, 2.85)	1.84(0.59, 3.50)	0.23	–
T-AL, mm	− 1.48(− 2.63, − 0.68)	− 2.54(− 3.50, − 1.40)	− 2.27(− 3.09, − 0.92)	0.24	–
T-AW, mm	− 0.30(− 0.95, 0.02)	− 0.45(− 1.31, 0.13)	− 0.12(− 1.24, 1.30)	0.37	–
T-MLA, mm	0.17(− 0.45, 0.63)	0.20(− 0.14, 0.74)	1.23(0.19, 1.83)	0.03*	(3,2) > (2,1)
T-ML, mm	− 0.89(− 1.06, − 0.47)	− 1.19(− 1.60, − 0.86)	− 1.48(− 1.73, − 1.14)	0.01*	(1,2) > (2,3)
T-MW, mm	− 0.55(− 0.88, − 0.12)	− 0.76(− 1.35, − 0.17)	− 0.19(− 0.99, 0.30)	0.25	–
VBL-LA, mm	0.55(− 0.02, 2.53)	0.01(− 0.68, 2.17)	0.06(− 0.24, 1.03)	0.27	–
VBL-L, mm	6.31(2.79, 7.54)	5.16(2.68, 7.22)	5.14(4.04, 7.10)	0.68	–
VBL-M, mm	0.45(0.08, 0.69)	0.08(− 0.42, 0.43)	0.28(0.05, 0.65)	0.10	–
VBL-D, mm	0.41(− 0.17, 0.56)	− 0.01(− 0.77, 0.61)	0.33(0.03, 0.89)	0.24	–

P, Mann–Whitney U test was performed for comparisons among group 1, group 2 and group 3; Results are expressed as median [interquartile range]

*P ≤ 0.05; **P ≤ 0.01

On the lingual side, during the decompensation course, the V-L, T-AL and T-ML of all three groups decreased significantly. At T2, the T-ML of all the groups was extremely thin (0.25 mm in the hyperdivergent group, 0.47 mm in the normodivergent group and 0.15 mm in the hypodivergent group on average) (Table 3). The ΔV-L and ΔT-ML of the hypodivergent group were significantly smaller than those of the hyperdivergent group, which meant that the hypodivergent group had more bone resorption on the lingual side in the retraction process (Table 5).

The VBL on the labial side of the hyperdivergent group increased by 1.53 mm on average, which meant that the labial alveolar bone height of the hypodivergent group decreased statistically, but was maintained in the normodivergent and hypodivergent groups. However, all three groups showed a significant increase in VBL on the lingual side, which meant a great reduction in lingual alveolar bone height. Although there was a slight increase in VBL-M and VBL-D in the hypodivergent group, the ΔVBL on the proximal sides of all three groups showed no apparent difference (Table 3).

The V-W and PDLA decreased significantly in all the groups, and the amount of reduction had no significant differences among the groups (Table 5). Although there was no significant change in the whole alveolar bone thickness at the root apex (T-AW) level during presurgical treatment for all the groups, the whole alveolar bone thickness at the middle root level (T-MW) of the hyperdivergent and normodivergent groups decreased significantly.

According to the results mentioned above, from T1 to T2, the labial alveolar bone volume and thickness were basically maintained or increased among groups; however, the lingual alveolar bone was significantly reduced. Therefore, in this study, groups, age, sex, ANB, SN-MP, IMPA, duration of therapy, measurements of alveolar bone at T1 and the amount of tooth movement were subjected to correlation analysis and stepwise regression analysis to censor variables that affected the 3D and 2D morphometric measurements of the lingual alveolar bone at T2, as shown in Table 6.

**Table 6:** Multiple linear regression analysis of lingual alveolar bone measurements at T2 of LCIs

Variables entered	*β*	Standard deviation	Standardized *β*	*P* values
*V-L (T2)*				
Δ S-apex	4.098	0.817	0.567	0.000**
T-AL (T1)	4.383	1.817	0.387	0.020*
V-L (T1)	0.249	0.119	0.318	0.043*
*T-AL (T2)*				
Δ S-apex	0.478	0.073	0.595	0.000**
T-AL (T1)	0.701	0.107	0.557	0.000**
Δ V-apex	0.349	0.074	0.38	0.000**
Δ AOL	− 0.045	0.018	− 0.219	0.014*
*VBL-L (T2)*				
T-ML (T1)	− 2.454	0.659	− 0.479	0.001**
Δ S-apex	− 0.673	0.19	− 0.456	0.001**

After Pearson correlation tests and multiple stepwise regression tests, equations with *P* values ≤ 0.05 were obtained, and the independent variables of interest were screened out

*P ≤ 0.05; **P ≤ 0.01

The regression analysis showed that on the lingual side, the V-L at T2 was positively influenced by V-L (T1), T-AL (T1) and ΔS-apex. The T-AL at T2 was positively influenced by ΔS-apex, T-AL (T1) and ΔV-apex, but negatively influenced by ΔAOL. A positive value of ΔAOL indicates proclination, and a negative value indicates retroclination. The VBL-L at T2 was negatively influenced by T-ML (T1) and ΔS-apex. Apart from the initial morphometric measurements at T1, the morphology of lingual alveolar bone at T2 was significantly influenced by root movement. Retraction and protrusion of the root apex and the decrease in Δ AOL were negatively related to the volume and thickness of alveolar bone on the lingual side at T2.

## Discussion

This study focuses mainly on quantifying the alveolar bone condition of lower incisors using the CBCT 3D technique during presurgical orthodontic treatment in adult skeletal Class II patients with different vertical skeletal patterns. Furthermore, our results reveal the detailed and comprehensive associations between the changes in the spatial position of the mandibular central incisors and the morphology of the lingual alveolar bone at different levels after the decompensation phase.

Before treatment, the morphology of the alveolar bone of the lower anterior teeth in different vertical patterns was different. For the hyperdivergent group, the alveolar bone thicknesses of the lingual side at the apical and midpoint levels were the thinnest among the groups [10, 14, 35], which suggested a greater lingual periodontal risk for LCIs during the root retracting process. To maintain the root within the alveolar bone and achieve the ideal overjet after decompensation for the desired surgical correction [36, 37] in the hyperdivergent group, the clinicians retracted LCIs in a controlled tipping manner with greater retraction of the crown than of the root apex (Fig. 3A). In contrast, the hypodivergent group had significantly greater lingual alveolar bone among the groups. Therefore, clinicians could retract LCIs of the hypodivergent group in a bodily manner with more torque control (Fig. 3C). Invasive alveolar bone resorption is a constant concern of orthodontists [19]. Our results provide insight into the effect of alveolar bone morphology on tooth movement in patients with different vertical patterns and remind clinicians to carefully consider periodontal condition and tooth movement in the treatment planning phase.

In the decompensation phase, for the hypodivergent and normodivergent groups, the labial alveolar bone volume increased or was maintained, and the VBLs at the labial sides were maintained. For the hyperdivergent group, although the labial alveolar bone thickness at the apical level increased, the alveolar bone height at the labial side decreased significantly, which was consistent with previous studies [5, 38]. The labial vertical bone loss in the hyperdivergent group could be partially explained by the result that the edge of the LCIs was slightly vertically extruded in the retraction process (Fig. 3A). Previous studies reported that the changes in labial alveolar bone remain controversial. After incisor retraction, some reported that the thickness of the labial alveolar bone at the apical level may be maintained or increase [5, 17, 39], while others reported a decrease in labial bone thickness at the cervical level [18, 40]. The differences in the enrolled patients in vertical skeletal patterns and retraction amount in these previous studies may explain the divergence in conclusions. In brief, for hyperdivergent patients, even if the labial alveolar bone volume was maintained, vertical marginal alveolar bone loss might occur at the labial side during the strong anchorage retracting process. Therefore, the risk of dehiscence, fenestration, gingival recession and black triangle on the labial side for hyperdivergent groups should not be ignored [41, 42]. Notably, for LCIs of hyperdivergent skeletal Class III malocclusion, apparent alveolar bone loss occurred at both labial and lingual sides in the LCI proclination process [21, 43]. Retraction or proclination movement is more likely to invade the mandibular alveolar bone barrier during orthodontic treatment, especially for the hyperdivergent group, as thinness of the labiolingual sides may be congenitally present in some patients. Labially augmented corticotomy-assisted orthodontics could provide a more favorable effect of improving periodontal status surrounding the mandibular anterior teeth for hyperdivergent patients [44].

The lingual alveolar bone volume, alveolar bone thickness and alveolar bone height of all three groups were significantly reduced during LCI retraction. After decompensation, the lingual bone thickness at the middle root level was extremely thin (< 1 mm on average) [45], and the vertical lingual alveolar bone height was reduced by more than 4 mm on average in each group, indicating that dehiscence occurred at the lingual side in all the groups [46], which suggested great periodontal risks and unoptimistic prognosis. Although before treatment, the lingual alveolar bone thickness of the hypodivergent group was significantly greater than that of the other groups, the lingual alveolar bone volume reduction of the hypodivergent group was significantly greater than that of the hyperdivergent group. The results suggested that in the case of strong anchorage retraction of LCIs, even if the lingual alveolar bone thickness of the hypodivergent group was greater before treatment, the risk of significant alveolar bone resorption and apparent dehiscence at the lingual side still existed because of the relatively large apical retraction. As a result, after decompensation, for all vertical skeletal malocclusion patterns, the periodontal risk of lingual alveolar bone should not be ignored during the retracting movement. Although a previous study [44] reported that with labial augmented corticotomy, the lingual thickness of the mandibular anterior teeth was reduced less than that of the control group after retraction movement, the loss of lingual vertical bone height of LCIs was statistically the same between the periodontal surgery group and the conventional group. Lu et al. [47] reported a single case of a Class I bialveolar protrusive malocclusion operating augmented corticotomy only on the lingual side in the mandibular anterior region. Their results showed that lingual augmented corticotomy could maintain periodontal health and increase the volume of soft and hard tissue. However, due to the small sample size and inadequate measurements, there was insufficient evidence to prove that this was directly related to the lingual augmented corticotomy operation. Therefore, safe and reliable lingual augmented corticotomy surgery still requires further research.

At present, bone remodeling during orthodontic treatment is still controversial. To avoid iatrogenic bone loss during orthodontic treatment, it is important to understand the bone remodeling ability of the patient and establish the amount of tooth movement prior to orthodontic treatment. The results of our study show that the morphology of lingual alveolar bone after decompensation was mainly affected by the initial condition of alveolar bone on the lingual side and the tooth movement patterns. The initial lingual alveolar bone volume and thickness were positively correlated with the volume and thickness of lingual alveolar bone after LCI retraction. Notably, the lingual bone height at T2 was correlated with the lingual thickness at the root midpoint level before treatment. The results suggested that with thinner lingual alveolar bone at the root midpoint level, the lingual marginal alveolar bone of LCIs would be more vulnerable to invasion during the retraction process, which may cause obvious bone dehiscence.

The tooth movement pattern also significantly affected the morphology of lingual alveolar bone after treatment. Greater horizontal apical retraction was significantly associated with greater lingual alveolar bone resorption [6, 25]. This study indicates that the retraction of LCIs with bodily movement was more susceptible to lingual alveolar bone recession than controlled tipping movement. In agreement with our study, Zhang et al. [48] and Hung et al. [6] also reported that bodily movement is more likely to influence the supporting alveolar bone. Furthermore, previous studies [49, 50] have reported that regarding proclined maxillary incisors, intrusion and retraction help reposition the teeth so that they are upright in the basal bone, leading to vertical alveolar bone gain. Our results showed similar findings in mandibular central incisors. The amount of vertical apex intrusion also had significant effects on lingual alveolar bone thickness after decompensation. Therefore, it can be hypothesized that intrusion of mandibular incisors may compensate for lingual alveolar bone loss during mandibular incisor retraction.

In our study, CBCT 3D measurements provided a more comprehensive understanding for clinicians regarding alveolar bone remodeling than 2D linear measurements. Our results showed that although there were no significant changes in the whole alveolar bone thickness at the apical level among the groups, the whole alveolar bone thickness at the root midpoint level decreased significantly. Hence, it is difficult to comprehensively represent the whole bone volume changes during treatment through 2D linear measurements such as bone thicknesses measured at specific CBCT levels. Nevertheless, 3D measurements, such as the whole alveolar bone volume and PDLA, were significantly reduced after the treatment, confirming that the total alveolar bone was reduced during the treatment. In addition, the digital 3D tooth and bone models used in this study could be saved as research material to investigate the specific sites of alveolar bone resorption for future studies. Therefore, 3D CBCT reconstruction provides useful information regarding periodontal defects and could be used as a complementary diagnostic technique to traditional 2D measurement.

Although this study provided informative findings on alveolar bone remodeling of LCIs for II malocclusions with different vertical skeletal patterns, limitations should be acknowledged. In this study, mandibular microimplants were used in all patients to achieve en masse retraction of LCIs. Hence, this study may not have fully reflected the effects of weak or moderate anchorage in the mandible. Moreover, the alveolar bone condition was evaluated within a very short period after finishing presurgical orthodontic treatment, and further research regarding long-term changes in alveolar bone is needed. Wang et al. [39] reported that after 18–24 months of retention, for LCIs, although the cervical alveolar bone seemed to recover over time to some extent, the alveolar bone condition did not reach the pretreatment levels. This indicates that the periodontal risk cannot be ignored even after long-term observation, which provides support for our study.

## Conclusion

In this study, we found that for Class II malocclusion patients undergoing presurgical orthodontic treatment, the changes in the periodontal support of LCIs varied in different vertical skeletal patterns. For hyperdivergent patients, vertical marginal alveolar bone loss might occur on the labial side. There exists a great periodontal risk of alveolar bone resorption on the lingual side for various vertical types. Furthermore, this study systematically investigated the correlation between the initial condition of alveolar bone, tooth movement patterns and alveolar bone morphology after presurgical orthodontics on the lingual side. By providing a more concrete understanding of their intercorrelation, this study could help orthodontists comprehensively consider the basic condition of periodontal support and adjust the movement types of LCIs to avoid undesirable alveolar bone resorption before treatment and have a relatively accurate prediction for periodontal prognosis after treatment. Additionally, 3D measurements based on CBCT construction can provide complementary information to traditional 2D measurements.

## Data Availability

The data set supporting the conclusions of this article is included within the article. Further data sets are available from the corresponding author on reasonable request.

## Abbreviations

CBCT: Cone-beam computed tomography
2D: Two-dimensional
3D: Three-dimensional
RL: Root length
LCI: Lower central incisor
VBL: Vertical alveolar bone level
ABT: Alveolar bone thickness
LA: Labial side
L: Lingual side
M: Mesial side
D: Distal side
SN plane: Sella–nasion plane
AOL: Angles of the long axes
DICOM: Digital Imaging and Communication in Medicine
STL: Stereolithographic
PDLA: Periodontal ligament area
T-ALA: Labial alveolar bone thickness at apex level
T-AL: Lingual alveolar bone thickness at apex level
T-AW: Whole alveolar bone thickness at apex level
T-MLA: Labial alveolar bone thickness at middle root level
T-ML: Lingual alveolar bone thickness at middle root level
T-MW: Whole alveolar bone thickness at middle root level
V-LA: Labial surrounding alveolar bone volume
V-L: Lingual surrounding alveolar bone volume
V-W: Whole surrounding alveolar bone volume
